# The Etiology of Pneumonia in HIV-1-infected South African Children in the Era of Antiretroviral Treatment: Findings From the Pneumonia Etiology Research for Child Health (PERCH) Study

**DOI:** 10.1097/INF.0000000000002651

**Published:** 2021-08-25

**Authors:** David P. Moore, Vicky L. Baillie, Azwifarwi Mudau, Jeannette Wadula, Tanja Adams, Shafeeka Mangera, Charl Verwey, Nosisa Sipambo, Afaaf Liberty, Christine Prosperi, Melissa M. Higdon, Meredith Haddix, Laura L. Hammitt, Daniel R. Feikin, Katherine L. O’Brien, Maria Deloria Knoll, David R. Murdoch, Eric A. F. Simões, Shabir A. Madhi

**Affiliations:** From the * South African Medical Research Council Vaccines and Infectious Diseases Analytics Research Unit, Faculty of Health Sciences, University of the Witwatersrand, Johannesburg, South Africa; † Department of Paediatrics & Child Health, Chris Hani Baragwanath Academic Hospital and University of the Witwatersrand, Johannesburg, South Africa; ‡ Department of Clinical Microbiology and Infectious Diseases, Chris Hani Baragwanath Academic Hospital, National Health Laboratory Service and University of the Witwatersrand, Johannesburg, South Africa; § Perinatal HIV Research Unit, Faculty of Health Sciences, University of the Witwatersrand, Johannesburg, South Africa; ¶ Department of International Health, International Vaccine Access Center, Johns Hopkins Bloomberg School of Public Health, Baltimore, MD; ‖ Department of Pathology, University of Otago, Christchurch, New Zealand; ** Microbiology Unit, Canterbury Health Laboratories, Christchurch, New Zealand; †† Department of Pediatrics, University of Colorado School of Medicine and Center for Global Health, Colorado School of Public Health, Aurora, CO.

**Keywords:** HIV-infected, pediatric, pneumonia, etiology, PERCH

## Abstract

HIV-1 infection predisposes to an increased burden of pneumonia caused by community-acquired and opportunistic pathogens.

**Methods:**

Within the context of the Pneumonia Etiology Research for Child Health case-control study of under 5 pneumonia, we investigated the etiology of World Health Organization-defined severe/very severe pneumonia requiring hospitalization in South African HIV-infected children. Nasopharyngeal-oropharyngeal swabs and blood, collected from cases and age- and season-matched HIV-infected controls attending outpatient antiretroviral therapy (ART) clinics, were analyzed using molecular diagnostic methods. Cases were also investigated for tuberculosis. Etiologic fractions among cases with radiologically confirmed pneumonia were derived using Bayesian analytic techniques.

**Results:**

Of 115 HIV-infected cases, 89 (77.4%) had radiologically confirmed pneumonia. Severe immunosuppression (adjusted odds ratio, 32.60; 95% confidence interval, 7.25–146.64) was significantly associated with radiologically confirmed pneumonia. Cotrimoxazole prophylaxis (46.4% vs. 77.4%) and ART (28.2% vs. 83.1%) coverage were significantly lower in cases compared with ART-clinic controls. An etiologic agent was identified in 99.0% of the radiologically confirmed cases. The ‘top 4’ pathogens associated with radiologically confirmed pneumonia were *Pneumocystis jirovecii* [23.0%; 95% credible interval (CrI), 12.4%–31.5%], *Staphylococcus aureus* (10.6%; 95% CrI, 2.2%–20.2%), pneumococcus (9.5%; 95% CrI, 2.2%–18.0%) and respiratory syncytial virus (9.3%; 95% CrI, 2.2%–14.6%). Bacteremia (6.7%) and in-hospital death (10.1%) were frequent among those with radiologically confirmed disease.

**Conclusions:**

*Pneumocystis jirovecii*, *S. aureus*, pneumococcus and respiratory syncytial virus contribute a considerable burden of radiologically confirmed pneumonia in South African HIV-infected children under 5 years. Expediting access to ART and cotrimoxazole prophylaxis would decrease the burden of pneumonia in these children.

## Abstract 2

Supplemental Digital Content is available in the text.

HIV-1 infection impacts on the etiology of pneumonia, including in South Africa where it is estimated that 400,000 (14%) of the 2.9 million HIV-infected sub-Saharan African children <15 years of age resided in 2012.^1^ Pneumonia is the leading cause of death in HIV-infected children,^2^ with respiratory pathogens associated with community-acquired pneumonia in HIV-uninfected children, and opportunistic pathogens, such as *Pneumocystis jirovecii*, human cytomegalovirus (CMV) and *Mycobacterium tuberculosis* (*Mtb*), causing a considerable burden of disease especially in young HIV-infected infants.^3^

Early peaks in mortality, occurring at 2 to 3 months of age, were noted to occur in South African infants prior to the national rollout of antiretroviral therapy (ART) for the treatment of HIV-infected persons in 2004.^4^ Most of these deaths were attributed to respiratory infections.^5^ These data suggest that pneumonia, which may have been caused by opportunistic pathogens, is common in young ART-naïve HIV-infected children.

Foundational studies of severe pneumonia etiology in South African HIV-infected children were undertaken in the mid-to-late 1990s,^6–9^ before polysaccharide-protein conjugate vaccines against *Haemophilus influenzae* type b (Hib) and *Streptococcus pneumoniae* were incorporated into the Expanded Program on Immunization. Furthermore, this was prior to the introduction of programs aimed at preventing vertical transmission of HIV from mother-to-child, and management of HIV-infected children with ART. These studies indicated that HIV-infected children had a higher incidence of bacterial, viral and opportunistic pneumonias than HIV-uninfected children, including higher case-fatality risk.^6–9^

The aim of this analysis was to describe the etiology of pneumonia in HIV-infected South African children in the era of access to ART and bacterial conjugate vaccines within the context of the Pneumonia Etiology Research for Child Health (PERCH) study. A companion paper^10^ describes pneumonia etiology in HIV-uninfected children at the South African PERCH site.

## MATERIALS AND METHODS

### Location

PERCH activities in South Africa took place at Chris Hani Baragwanath Academic Hospital (CHBAH), situated in Soweto, the most populous township in Johannesburg, Gauteng Province. Although South Africa is classified as an upper middle-income country, it is ranked among the most unequal societies economically.^11,12^ At the time of the study, the unemployment rate in Soweto, an urban low-income community, was 32% and 40% of households survived on <2 USD per day.^13,14^

Health care in South Africa is provided at no cost to the family for all children <6 years of age who utilize the public health sector.^15^ Hib conjugate vaccine (HibCV) and pneumococcal conjugate vaccine (PCV) were introduced into the South African Expanded Program on Immunization in 1999 and 2009, respectively. World Health Organization (WHO) estimates of coverage with a third dose of HibCV and PCV in South Africa were 69% and 75%, respectively, in 2012.^16^

The respiratory virus season in Soweto, including circulation of respiratory syncytial virus (RSV) and influenza virus, generally occurs in the autumn/winter months of March through August.^17,18^

The antenatal HIV prevalence in Soweto remained stable at 29.0% and 27.3% in 2009 and 2013, respectively,^19^ while vertical transmission of HIV from mother-to-child declined from 9.6% in 2008 to 2.2% by 2012/2013.^20,21^ The national ART program achieved 45.1% coverage of an estimated 369,000 HIV-infected children <14 years of age in need of ART by 2012.^22^ Eligibility criteria for ART in children, and ART regimens used, are described in Supplemental Digital Content 1, http://links.lww.com/INF/D843.

### Participants

For this analysis, cases were HIV-infected children between the ages of one and 59 months, hospitalized with signs of WHO-defined severe or very severe pneumonia (according to the 2005 case definitions)^23^ and resident in the study catchment area. The study catchment area consisted of the area from which 90% of PERCH age-eligible children hospitalized at CHBAH resided in 2010, the year prior to the start of PERCH enrollment at the South African site. ART-clinic controls, resident in the study catchment area and attending 2 pediatric HIV Clinics at CHBAH as outpatients, were frequency-matched to cases according to age-stratification (1–5, 6–11, 12–23 and 24–59 months) (see Supplemental Digital Content 1, http://links.lww.com/INF/D843).

### Clinical Procedures

Case enrollment occurred through active surveillance in the hospital pediatric admissions ward. Cases were evaluated at enrollment, 24 and 48 hours thereafter, on the day of hospital discharge, and (if surviving to hospital discharge) at 30 days post-admission to evaluate vital status. Case 30-day vital assessments were done at the research unit outpatient clinic, whilst control enrollment clinical assessments were done at outpatient ART clinics on the hospital premises.

### Specimen Collection and Laboratory Methods

Blood and respiratory specimens were collected in a standardized manner from cases and controls as described,^24–27^ and chest radiographs were obtained from cases as soon as possible after hospitalization.^28^ Comparator specimens used to evaluate the microbiologic milieu in cases and controls consisted of nasopharyngeal-oropharyngeal (NP/OP) swabs, for real-time multiplex polymerase chain reaction (PCR) to detect 33 respiratory pathogens (Fast Track Diagnostics Respiratory Pathogens 33 test, Fast Track Diagnostics, Sliema, Malta) and whole blood for pneumococcal autolysin (*lytA*) PCR. Specimen collection procedures, including age-appropriate HIV testing with confirmation of HIV-infection status, are outlined in Supplemental Digital Content 1, http://links.lww.com/INF/D843.

### Statistical Analysis

Descriptive analyses for clinical and laboratory measures were done, reporting percentages in subgroups (cases stratified by chest radiographic findings, pneumonia severity and in-hospital mortality) and comparing proportions using logistic regression, adjusting for age (in months) and season. Continuous variables were summarized as medians and interquartile ranges (IQRs). When multiple comparisons were done, *P* values were adjusted using the Benjamini-Hochberg method.^29^ Two-sided *P* values <0.05 were considered to be statistically significant.

Organism density cutoff values which best distinguished between case and ART-clinic control status on NP/OP swabs and whole blood (*lytA*) were derived as part of the PERCH foundational analyses^30–33^ and were applied to *S. pneumoniae* (as detected in NP/OP swabs and whole blood) and CMV, *H. influenzae* and *P. jirovecii* (as detected in NP/OP swabs) in the current analysis. Conditional odds ratios of pathogen prevalence in cases and controls were derived using logistic regression, adjusting for age in months and all other pathogens. As pneumococcus was tested for in NP/OP swabs and whole blood, it was reciprocally excluded depending on which pneumococcal test was evaluated in the logistic regression model, for example, if *LytA* positivity was the focus of the analysis, pneumococcal NP/OP results were excluded from the model. Data were integrated using a Bayesian approach capable of generating site-specific pneumonia etiology profiles from a variety of specimens and tests done on cases and controls, as previously described.^34,35^ We also estimated the proportion of cases with no identifiable pathogen. Bayesian analytic outputs were informed, but not governed, by the conditional logistic regression analyses. An open-source R software package called the Bayesian Analysis Kit for Etiology Research, which is available at https://github.com/zhenkewu/baker, was developed for the PERCH Integrated Analysis (PIA). The PIA is a single-etiology model which did not evaluate for pathogen-pathogen interactions in the pneumonia etiologic process. Further detail on the PIA is included in Supplemental Digital Content 1, http://links.lww.com/INF/D843. Analyses were performed using R version 3.3.3,^36^ SAS 9.4 (SAS Institute, Cary, NC) and JAGS 4.2.0 (http://mcmc-jags.sourceforge.net/).

The sensitivity prior for *Mtb* in the South African site-specific etiologic analyses presented here and in the HIV-uninfected cohort^10^ was set at 20% to 50% rather than the 10% to 30% sensitivity prior used to estimate the etiologic fraction (EF) of *Mtb* at other PERCH sites, and for the South African site in the all-site PERCH paper.^37^ The higher sensitivity prior was chosen in view of South Africa being a high-burden setting of HIV and tuberculosis, and because there was a more extensive diagnostic work-up for tuberculosis in South African PERCH cases. *Mtb* cultured on endotracheal tube aspirates are described but were not included as a positive measurement in the PIA.

### Ethical Considerations

We obtained written informed consent for participation in the study from parents or legal guardians of all participants. The study was approved by the Human Research Ethics Committee of the University of the Witwatersrand (M101129) and the Institutional Review Board of the Johns Hopkins Bloomberg School of Public Health.

## RESULTS

### Study Participants

A total of 920 severe pneumonia cases and 964 controls were enrolled between 17 August 2011 and 4 September 2013 at the South African PERCH site, of whom 115 (12.5%) cases and 136 (14.1%) controls were HIV-infected (Supplemental Digital Content 2, http://links.lww.com/INF/D844). All but 2 of the HIV-infected controls were enrolled at the hospital’s pediatric ART clinics. The remainder of the analysis presented herein is focused on the HIV-infected children.

The median age of HIV-infected participants was 7.0 months (IQR, 3.0–13.5 months) in cases and 9.5 months (IQR, 4.5–20.5 months) among ART-clinic controls; *P* = 0.118. Cases had more advanced disease compared with ART-clinic controls, as demonstrated by their degree of immunosuppression (71.2% cases vs. 34.6% controls had severe immunosuppression) and WHO clinical stage of disease (70.4% cases vs. 37.5% controls had WHO Stage IV disease) (Table 1). Cases were also more likely to be severely under-weight-for age (32.5% vs. 12.6%) (Table 1). There was similar immunization coverage with bacillus Calmette-Guérin (96.2% in cases vs. 96.7% in ART-clinic controls) and PCV (67.9% in cases vs. 62.6% in ART-clinic controls) (Supplemental Digital Content 3, http://links.lww.com/INF/D845). ART and cotrimoxazole coverage was significantly lower in cases compared with ART-clinic controls, however; *P* < 0.001 for both comparisons (Table 1).

**TABLE 1.** Demographic and Clinical Characteristics of HIV-infected Cases and ART-clinic Controls Enrolled Into PERCH at the South African Site

Characteristic	All Cases (n = 115)	CXR + Cases (n = 89/112)	ART-clinic Controls (n = 136)	OR (95% CI); Adjusted *P* Value*	
				All Cases Compared with ART-clinic Controls	CXR + Cases Compared with ART-clinic Controls
Age (months)					
Median age (IQR)	7.0 (3.0–13.5)	7.0 (3.0–14.0)	9.5 (4.5–20.5)	0.98 (0.96–1.00); 0.121	0.98 (0.96–1.00); 0.152
Sex					
Female	57/115 (49.6)	42/89 (47.2)	78/136 (57.4)	0.69 (0.42–1.16); 0.291	0.61 (0.35–1.06); 0.152
Respiratory tract illness (controls only)†	-	-	6/136 (4.4)	-	-
Anthropometry					
WAZ ≥−2	55/114 (48.2)	40/88 (45.5)	96/135 (71.1)	Ref	Ref
WAZ ≥−3 to <−2	22/114 (19.3)	18/88 (20.5)	22/135 (16.3)	1.67 (0.84–3.32); 0.259	1.92 (0.92–4.00); 0.152
WAZ <−3	37/114 (32.5)	30/88 (34.1)	17/135 (12.6)	3.66 (1.87–7.17); <0.001	4.14 (2.03–8.41); <0.001
CRP					
Median, mg/L (IQR)	21.4 (5.0–105.8)	26.4 (5.8–132.5)	1.3 (0.4–2.2)	1.05 (1.01–1.09); 0.071	1.05 (1.00–1.10); 0.068
≥40, mg/L	39/112 (34.8)	32/86 (37.2)	1/27 (3.7)	16.18 (1.88–138.96); 0.032	16.20 (1.91–137.45); 0.031
Prior exposure to medications					
Serum antibiotic activity	64/110 (58.2)	46/84 (54.8)	16/118 (13.6)	8.23 (4.25–15.93); <0.001	7.26 (3.64–14.51); <0.001
Cotrimoxazole prophylaxis	42/86 (48.8)	32/69 (46.4)	103/133 (77.4)	0.27 (0.15–0.49); <0.001	0.24 (0.12–0.45); <0.001
ART-clinic characteristics					
ART-clinic attendance‡	28/49 (57.1)	21/36 (58.3)	117/134 (87.3)	0.17 (0.07–0.38); <0.001	0.19 (0.08–0.44); <0.001
On ART at PERCH enrollment	33/110 (30.0)	24/85 (28.2)	113/136 (83.1)	0.09 (0.05–0.16); <0.001	0.08 (0.04–0.16); <0.001
Median time on ART (weeks, IQR)	0.0 (0.0–10.0)	0.0 (0.0–5.4)	14.6 (4.7–40.6)	0.99 (0.98–0.99); 0.008	0.98 (0.96–0.99); <0.001
CD4 absolute count§	808 (440–1372)	773 (442–1319)	1725 (1176–2328)	0.9989 (0.9985–0.9992); <0.001	0.9987 (0.9983–0.9992); <0.001
CD4 %	14.9 (8.7–23.8)	14.0 (8.5–22.9)	27.4 (21.7–34.3)	0.91 (0.89–0.94); <0.001	0.90 (0.87–0.93); <0.001
Degree of immunosuppression¶					
None	6/111 (5.4)	2/86 (2.3)	47/133 (35.3)	Ref	Ref
Mild	13/111 (11.7)	11/86 (12.8)	17/133 (12.8)	5.64 (1.81–17.58); 0.012	15.44 (3.01–79.25); 0.004
Advanced	13/111 (11.7)	11/86 (12.8)	23/133 (17.3)	4.24 (1.40–12.88); 0.032	11.57 (2.30–58.18); 0.011
Severe	79/111 (71.2)	62/86 (72.1)	46/133 (34.6)	12.72 (4.90–33.00); <0.001	32.60 (7.25–146.64); <0.001
WHO Clinical Stage					
I	3/115 (2.6)	1/89 (1.1)	60/136 (44.1)	Ref	Ref
II	3/115 (2.6)	2/89 (2.2)	3/136 (2.2)	18.19 (2.44–135.55); 0.017	37.17 (2.51–551.19); 0.026
III	28/115 (24.3)	21/89 (23.6)	22/136 (16.2)	31.57 (8.53–116.77); <0.001	71.97 (9.00–575.48); <0.001
IV	81/115 (70.4)	65/89 (73.0)	51/136 (37.5)	38.80 (11.37–132.40); <0.001	93.09 (12.35–701.58); <0.001

*Odds ratio adjusted by age (in months) and season, and derived by logistic regression analysis. *P* values adjusted using the Benjamini-Hochberg method.

†Respiratory tract illness in PERCH controls was defined as presence of cough or runny nose, or if a child had (1) at least 1 of ear discharge, wheezing, or difficulty breathing and (2) either a measured temperature of >38.0°C within the previous 48 hours or a history of sore throat.

‡ART-clinic attendance in the preceding 3 months.

§Cells × 10^6^/L.

¶Immunosuppression categorized according to the World Health Organization system.

CI indicates confidence interval; CRP, C-reactive protein; CXR+, radiologically confirmed pneumonia; Ref, referent; WAZ, weight-for-age Z-score.

### Case Characteristics

Thirty-four percent (39/115) of cases had very severe pneumonia, and 89 (79.5%) of 112 with an available chest radiograph had confluent alveolar consolidation and/or other infiltrates. The in-hospital case-fatality ratio among HIV-infected cases with radiologically confirmed pneumonia was 10.1% (9/89), and all 6 children dying subsequent to hospital discharge but before the 30-day follow-up vital assessment also had radiologically confirmed pneumonia (Supplemental Digital Content 4, http://links.lww.com/INF/D846). After adjusting for age and multiple comparisons, no clinical or laboratory feature was significantly associated with clinical pneumonia severity, abnormal chest radiographic findings or in-hospital death (data not shown).

### Microbiologic Results in HIV-infected Cases

Blood cultures were available from all cases, and one case underwent lung aspirate sampling. Nine (7.8%) of the blood cultures yielded a significant pathogen, while 15 (13.0%) identified a presumed contaminant. Gram-negative organisms constituted two-thirds (6 of 9) of the significant blood cultured pathogens (Supplemental Digital Content 5, http://links.lww.com/INF/D847). Six (6.7%) of the 89 children with radiologically confirmed pneumonia cultured clinically significant bacteria on blood culture. There were three microbiologically confirmed pneumococcal cases all of whom had radiologically confirmed pneumonia, including serotype 16F on blood culture in a 10-month old incompletely PCV-vaccinated female with WHO stage 4 HIV disease; pneumococcus identified by latex antigen positivity on a flag-positive, culture-negative blood culture in a 58-month-old male with undetermined pneumococcal vaccination status; and pneumococcus identified by PCR of lung aspirate material in an 8-month old female who had received 2 primary doses of PCV.

*Mtb* was cultured in 3 (2.6%) of the 115 cases investigated, on gastric aspirate samples in 2 (2.5%) of 80 children and endotracheal tube aspirate from 1 (14.3%) of 7 cases. None of the induced sputum samples (available from 100 cases) yielded *Mtb* on culture. Seven of 65 (10.8%) cases had a positive tuberculin skin test (≥5 mm induration) (Supplemental Digital Content 5, http://links.lww.com/INF/D847). One of the 7 children with a positive tuberculin skin test cultured *Mtb*, on the third gastric aspirate specimen.

### Comparison of NP/OP Fast Track Diagnostics Respiratory Pathogens 33 and Whole Blood LytA PCR Results Between HIV-infected Cases and Controls

HIV-infected cases with radiologically confirmed pneumonia had significantly higher age-adjusted odds of any nonviral organism detected above cutoff density thresholds on NP/OP swabs than did controls [87.6% vs. 71.3%, adjusted odds ratio (aOR), 2.80; Table 2]. Specifically, *Staphylococcus aureus* (47.2% in cases; aOR, 2.49) and *P. jirovecii* (27.0% above threshold density in cases; aOR, 19.11) were detected more frequently in cases with radiologically confirmed pneumonia compared with controls (Table 2).

**TABLE 2.** Conditional Odds Ratios in the Comparison Between All Cases, Cases With Radiologically Confirmed Pneumonia, and ART-clinic Controls: HIV-infected Children

Pathogen	All Cases	CXR + Cases	ART-clinic Controls	Conditional Odds Ratio (95% CI) *	
				All Cases vs. ART-clinic Controls	CXR + Cases vs. ART-clinic Controls
Any nonviral pathogen	105/115 (91.3)	81/89 (91.0)	115/136 (84.6)	1.69 (0.73–3.96)	1.68 (0.67–4.19)
Any nonviral pathogen, above cutoff density threshold†	101/115 (87.8)	78/89 (87.6)	97/136 (71.3)	2.82 (1.41–5.65)	2.80 (1.31–5.97)
Bacteria					
*Bordetella pertussis*	1/115 (0.9)	1/89 (1.1)	0/136 (0.0)	N/E	N/E
*Chlamydophila pneumoniae*	0/115 (0.0)	0/89 (0.0)	0/136 (0.0)	N/E	N/E
*Haemophilus influenzae* type b	1/115 (0.9)	1/89 (1.1)	2/136 (1.5)	0.76 (0.06–10.43)	0.88 (0.06–12.50)
*Haemophilus influenzae* type b ≥ threshold density‡	0/115 (0.0)	0/89 (0.0)	0/136 (0.0)	N/E	N/E
Nontype b *Haemophilus influenzae*	62/115 (53.9)	50/89 (56.2)	50/136 (36.8)	1.80 (0.91–3.57)	1.74 (0.81–3.71)
Nontype b *Haemophilus influenzae* ≥ threshold density‡	42/115 (36.5)	33/89 (37.1)	26/136 (19.1)	2.03 (0.93–4.40)	1.99 (0.84–4.72)
*Moraxella catarrhalis*	70/115 (60.9)	57/89 (64.0)	66/136 (48.5)	1.23 (0.61–2.48)	1.49 (0.68–3.25)
*Mycoplasma pneumoniae*	0/115 (0.0)	0/89 (0.0)	1/136 (0.7)	N/E	N/E
*Streptococcus pneumoniae*	75/115 (65.2)	58/89 (65.2)	81/136 (59.6)	1.37 (0.64–2.93)	1.26 (0.54–2.90)
*Streptococcus pneumoniae* ≥ threshold density§	22/115 (19.1)	20/89 (22.5)	15/136 (11.0)	1.21 (0.46–3.20)	1.46 (0.51–4.15)
Vaccine-type *Streptococcus pneumoniae*¶	13/115 (11.3)	12/89 (13.5)	6/136 (4.4)	1.91 (0.53–6.84)	2.53 (0.65–9.93)
Nonvaccine-type *Streptococcus pneumoniae*¶	9/115 (7.8)	8/89 (9.0)	9/136 (6.6)	0.59 (0.16–2.22)	0.60 (0.15–2.51)
*Streptococcus pneumoniae* in whole blood	14/115 (12.2)	13/89 (14.6)	12/136 (8.8)	1.62 (0.55–4.82)	1.96 (0.61–6.37)
*Streptococcus pneumoniae* in whole blood ≥ threshold density‖	11/115 (9.6)	11/89 (12.4)	7/136 (5.1)	1.93 (0.53–7.02)	2.37 (0.62–9.05)
Salmonella spp	0/115 (0.0)	0/89 (0.0)	1/136 (0.7)	N/E	N/E
*Staphylococcus aureus*	52/115 (45.2)	42/89 (47.2)	28/136 (20.6)	2.27 (1.12–4.63)	2.49 (1.15–5.40)
Fungal species					
*Pneumocystis jirovecii*	38/115 (33.0)	28/89 (31.5)	8/136 (5.9)	8.67 (3.39–22.17)	7.76 (2.81–21.46)
*Pneumocystis jirovecii* ≥ threshold density**	33/115 (28.7)	24/89 (27.0)	4/136 (2.9)	18.76 (5.66–62.20)	19.11 (5.20–70.24)
Viruses					
Any viral pathogen	106/115 (92.2)	83/89 (93.3)	96/136 (70.6)	4.35 (1.98–9.58)	5.15 (2.05–12.94)
Any viral pathogen, above cutoff density threshold†	94/115 (81.7)	73/89 (82.0)	87/136 (64.0)	2.10 (1.14–3.87)	2.12 (1.09–4.15)
Adenovirus	20/115 (17.4)	17/89 (19.1)	13/136 (9.6)	1.87 (0.72–4.91)	1.74 (0.63–4.80)
Human cytomegalovirus	85/115 (73.9)	71/89 (79.8)	74/136 (54.4)	2.00 (0.97–4.10)	2.66 (1.15–6.13)
Human cytomegalovirus ≥ threshold density††	59/115 (51.3)	49/89 (55.1)	41/136 (30.1)	2.52 (1.26–5.06)	3.38 (1.54–7.43)
Coronavirus 229	0/115 (0.0)	0/89 (0.0)	0/136 (0.0)	N/E	N/E
Coronavirus 43	3/115 (2.6)	3/89 (3.4)	9/136 (6.6)	0.49 (0.07–3.70)	0.70 (0.09–5.60)
Coronavirus 63	2/115 (1.7)	2/89 (2.2)	1/136 (0.7)	1.82 (0.12–28.40)	2.50 (0.15–41.06)
Coronavirus HKU	0/115 (0.0)	0/89 (0.0)	5/136 (3.7)	N/E	N/E
Influenza A	2/115 (1.7)	1/89 (1.1)	0/136 (0.0)	N/E	N/E
Influenza B	0/115 (0.0)	0/89 (0.0)	0/136 (0.0)	N/E	N/E
Influenza C	1/115 (0.9)	1/89 (1.1)	1/136 (0.7)	1.01 (0.03–33.02)	1.86 (0.06–57.45)
Human bocavirus	18/115 (15.7)	15/89 (16.9)	14/136 (10.3)	1.84 (0.67–5.08)	1.86 (0.62–5.57)
Human metapneumovirus A/B	5/115 (4.3)	4/89 (4.5)	3/136 (2.2)	1.10 (0.18–6.75)	1.24 (0.16–9.35)
Parainfluenza virus 1	6/115 (5.2)	3/89 (3.4)	3/136 (2.2)	2.60 (0.44–15.22)	1.12 (0.13–10.07)
Parainfluenza virus 2	1/115 (0.9)	1/89 (1.1)	3/136 (2.2)	0.64 (0.05–7.94)	1.09 (0.08–14.04)
Parainfluenza virus 3	11/115 (9.6)	11/89 (12.4)	4/136 (2.9)	3.87 (0.98–15.37)	5.71 (1.38–23.58)
Parainfluenza virus 4	1/115 (0.9)	1/89 (1.1)	2/136 (1.5)	1.06 (0.08–14.15)	1.46 (0.10–20.81)
Parechovirus/Enterovirus	3/115 (2.6)	2/89 (2.2)	11/136 (8.1)	0.83 (0.19–3.61)	0.70 (0.12–3.92)
Human rhinovirus	28/115 (24.3)	19/89 (21.3)	18/136 (13.2)	2.19 (0.93–5.13)	1.93 (0.74–5.04)
Respiratory syncytial virus	12/115 (10.4)	11/89 (12.4)	3/136 (2.2)	13.74 (2.51–75.20)	15.42 (2.67–88.93)

*Conditional odds ratio derived by logistic regression, adjusting age (in months) and presence of all other pathogens. Two analyses were combined in the output of this Table: the first with no threshold applied for human cytomegalovirus, *H. influenzae*, *P. jirovecii*, and *S. pneumoniae*, and the second with threshold density cutoffs (as noted below) applied to these pathogens. The first analysis output was used to report the adjusted conditional odds for cytomegalovirus, *H. influenzae*, *P. jirovecii*, and *S. pneumoniae* with no threshold density cutoff applied. The second analysis output was used to report the adjusted conditional odds for all pathogen named in the table.

†Cutoff density threshold which best distinguished between cases and controls, derived by receiver operating characteristic analysis using leave-one-out cross-validation. For viral pathogens, a threshold density cutoff was applied to human cytomegalovirus only.

‡Cutoff density for *H. influenzae* (nontype b, and type b) on NP/OP swabs: 5.9 log_10_ copies/mL.

§Cutoff density for *S. pneumoniae* on NP/OP swabs: 6.9 log_10_ copies/mL.

¶Vaccine-type pneumococcus among children with high density NP/OP pneumococcal carriage.

‖Cutoff density for *S. pneumoniae* in whole blood specimens: 2.2 log_10_ copies/mL.

**Cutoff density for *P. jirovecii* on NP/OP swabs: 4.0 log_10_ copies/mL.

††Cutoff density for human cytomegalovirus on NP/OP swabs: 4.9 log_10_ copies/mL.

CI indicates confidence interval; CXR+, radiologically confirmed pneumonia; N/E, no estimate; NP/OP, nasopharyngeal/oropharyngeal.

The pooled analysis of viral pathogens indicated that these pathogens were significantly more prevalent in HIV-infected cases with radiologically confirmed pneumonia compared with controls but less so when the threshold density cutoff for CMV was applied (Table 2). Of the viral pathogens CMV (55.1% above threshold density in cases; aOR, 3.38), parainfluenza virus 3 (12.4% in cases; aOR, 5.71) and RSV (12.4% in cases; aOR, 15.42) were detected significantly more frequently in cases with radiologically confirmed pneumonia compared with controls (Table 2).

After adjusting for age (in months) and all other pathogens, organisms associated with in-hospital mortality in HIV-infected children included adenovirus, *P. jirovecii* (as detected on NP/OP swabs) and pneumococcus (as detected on whole blood PCR) (Table 3).

**TABLE 3.** Conditional Odds Ratios in the Comparison Between Cases Dying In-hospital and Cases Surviving to Hospital Discharge, and ART-clinic Controls: HIV-infected Children

Pathogen	Cases Dying In-hospital	Cases Surviving to Hospital Discharge	ART-clinic Controls	Conditional Odds Ratio (95% CI)*	
				Cases Dying In-hospital vs. Cases Surviving to Hospital Discharge	Cases Dying In-hospital vs. ART-clinic Controls
Any nonviral pathogen	14/17 (82.4)	91/98 (92.9)	115/136 (84.6)	0.34 (0.08–1.53)	0.81 (0.20–3.30)
Any nonviral pathogen, above cutoff density threshold†	14/17 (82.4)	87/98 (88.8)	97/136 (71.3)	0.60 (0.15–2.49)	1.78 (0.46–6.89)
Bacteria					
*Bordetella pertussis*	0/17 (0.0)	1/98 (1.0)	0/136 (0.0)	N/E	N/E
*Chlamydophila pneumoniae*	0/17 (0.0)	0/98 (0.0)	0/136 (0.0)	N/E	N/E
*Haemophilus influenzae* type b	0/17 (0.0)	1/98 (1.0)	2/136 (1.5)	N/E	N/E
*Haemophilus influenzae* type b ≥ threshold density‡	0/17 (0.0)	0/98 (0.0)	0/136 (0.0)	N/E	N/E
Nontype b *Haemophilus influenzae*	7/17 (41.2)	55/98 (56.1)	50/136 (36.8)	0.89 (0.20–4.00)	1.75 (0.33–9.14)
Nontype b *Haemophilus influenzae* ≥ threshold density‡	5/17 (29.4)	37/98 (37.8)	26/136 (19.1)	0.69 (0.14–3.39)	1.84 (0.13–26.62)
*Moraxella catarrhalis*	10/17 (58.8)	60/98 (61.2)	66/136 (48.5)	0.99 (0.26–3.84)	0.68 (0.10–4.83)
*Mycoplasma pneumoniae*	0/17 (0.0)	0/98 (0.0)	1/136 (0.7)	N/E	N/E
*Streptococcus pneumoniae*	9/17 (52.9)	66/98 (67.3)	81/136 (59.6)	0.53 (0.11–2.48)	0.85 (0.13–5.45)
*Streptococcus pneumoniae* ≥ threshold density§	3/17 (17.6)	19/98 (19.4)	15/136 (11.0)	1.41 (0.25–8.12)	7.79 (0.46–131.18)
Vaccine-type *Streptococcus pneumoniae*¶	1/17 (5.9)	12/98 (12.2)	6/136 (4.4)	0.46 (0.04–5.77)	2.31 (0.03–175.43)
Nonvaccine-type *Streptococcus pneumoniae*¶	2/17 (11.8)	7/98 (7.1)	9/136 (6.6)	4.00 (0.43–37.25)	16.93 (0.58–495.59)
*Streptococcus pneumoniae* in whole blood	1/17 (5.9)	13/98 (13.3)	12/136 (8.8)	1.23 (0.10–14.94)	9.93 (0.55–180.85)
*Streptococcus pneumoniae* in whole blood ≥ threshold density‖	1/17 (5.9)	10/98 (10.2)	7/136 (5.1)	2.24 (0.15–33.19)	62.83 (1.69–2341.31)
Salmonella spp	0/17 (0.0)	0/98 (0.0)	1/136 (0.7)	N/E	N/E
*Staphylococcus aureus*	8/17 (47.1)	44/98 (44.9)	28/136 (20.6)	1.30 (0.31–5.46)	0.45 (0.05–3.85)
Fungal species					
*Pneumocystis jirovecii*	10/17 (58.8)	28/98 (28.6)	8/136 (5.9)	3.83 (0.72–20.40)	23.12 (3.75–142.40)
*Pneumocystis jirovecii* ≥ threshold density**	10/17 (58.8)	23/98 (23.5)	4/136 (2.9)	7.17 (1.28–40.28)	126.49 (10.02–1604.33)
Viruses					
Any viral pathogen	16/17 (94.1)	90/98 (91.8)	96/136 (70.6)	1.27 (0.14–11.80)	6.48 (0.80–52.52)
Any viral pathogen, above cutoff density threshold†	15/17 (88.2)	79/98 (80.6)	87/136 (64.0)	1.65 (0.33–8.17)	3.58 (0.76–16.91)
Adenovirus	3/17 (17.6)	17/98 (17.3)	13/136 (9.6)	1.65 (0.29–9.41)	17.60 (1.32–234.92)
Human cytomegalovirus	13/17 (76.5)	72/98 (73.5)	74/136 (54.4)	1.78 (0.34–9.17)	2.30 (0.39–13.55)
Human cytomegalovirus ≥ threshold density††	10/17 (58.8)	49/98 (50.0)	41/136 (30.1)	1.78 (0.46–6.84)	2.85 (0.45–18.00)
Coronavirus 229	0/17 (0.0)	0/98 (0.0)	0/136 (0.0)	N/E	N/E
Coronavirus 43	0/17 (0.0)	3/98 (3.1)	9/136 (6.6)	N/E	N/E
Coronavirus 63	0/17 (0.0)	2/98 (2.0)	1/136 (0.7)	N/E	N/E
Coronavirus HKU	0/17 (0.0)	0/98 (0.0)	5/136 (3.7)	N/E	N/E
Influenza A	1/17 (5.9)	1/98 (1.0)	0/136 (0.0)	38.25 (0.56–2591.54)	N/E
Influenza B	0/17 (0.0)	0/98 (0.0)	0/136 (0.0)	N/E	N/E
Influenza C	1/17 (5.9)	0/98 (0.0)	1/136 (0.7)	N/E	176.01 (0.05–597435.14)
Human bocavirus	1/17 (5.9)	17/98 (17.3)	14/136 (10.3)	0.55 (0.04–8.14)	1.36 (0.09–21.28)
Human metapneumovirus A/B	0/17 (0.0)	5/98 (5.1)	3/136 (2.2)	N/E	N/E
Parainfluenza virus 1	3/17 (17.6)	3/98 (3.1)	3/136 (2.2)	3.45 (0.30–39.47)	7.80 (0.04–1610.14)
Parainfluenza virus 2	0/17 (0.0)	1/98 (1.0)	3/136 (2.2)	N/E	N/E
Parainfluenza virus 3	2/17 (11.8)	9/98 (9.2)	4/136 (2.9)	1.20 (0.10–14.94)	0.60 (0.02–19.25)
Parainfluenza virus 4	0/17 (0.0)	1/98 (1.0)	2/136 (1.5)	N/E	N/E
Parechovirus/Enterovirus	1/17 (5.9)	2/98 (2.0)	11/136 (8.1)	23.01 (0.70–750.83)	3.98 (0.22–72.13)
Human rhinovirus	4/17 (23.5)	24/98 (24.5)	18/136 (13.2)	1.45 (0.29–7.24)	2.24 (0.20–25.08)
Respiratory syncytial virus	0/17 (0.0)	12/98 (12.2)	3/136 (2.2)	N/E	N/E

* Conditional odds ratio derived by logistic regression, adjusting age (in months) and presence of all other pathogens: two analyses were combined in the output of this table: the first with no threshold applied for human cytomegalovirus, *H. influenzae*, *P. jirovecii*, and *S. pneumoniae*, and the second with threshold density cutoffs (as noted below) applied to these pathogens. The first analysis output was used to report the adjusted conditional odds for cytomegalovirus, *H. influenzae*, *P. jirovecii*, and *S. pneumoniae* with no threshold density cutoff applied. The second analysis output was used to report the adjusted conditional odds for all pathogens named in the table.

†Cutoff density threshold which best distinguished between cases and controls, derived by receiver operating characteristic analysis using leave-one-out cross-validation.

‡Cutoff density for *H. influenzae* (nontype b, and type b) on NP/OP swabs: 5.9 log_10_ copies/mL.

§Cutoff density for *S. pneumoniae* on NP/OP swabs: 6.9 log_10_ copies/mL.

¶Vaccine-type pneumococcus among children with high density NP/OP pneumococcal carriage.

‖Cutoff density for *S. pneumoniae* in whole blood specimens: 2.2 log_10_ copies/mL.

**Cutoff density for *P. jirovecii* on NP/OP swabs: 4.0 log_10_ copies/mL.

††Cutoff density for human cytomegalovirus on NP/OP swabs: 4.9 log_10_ copies/mL.

CI indicates confidence interval; CXR+, radiologically confirmed pneumonia; N/E, no estimate; NP/OP, nasopharyngeal/oropharyngeal.

### PIA Determination of Pathogen Etiology Fraction in HIV-infected South African Children

Bayesian analytic outputs indicated that *P. jirovecii* [EF 23.0%; 95% credible interval (CrI), 12.4%–31.5%] was the top-ranked organism associated with case-status in HIV-infected children with radiologically confirmed pneumonia. Other top pathogens included *S. aureus* (EF 10.6%), *S. pneumoniae* (EF 9.5%) and RSV (EF 9.3%) (Figure). The EFs attributed to *Mtb* and CMV were 4.7% and 1.9%, respectively (Figure). No identified pathogen could be assigned to case-status in 1.0% (95% CrI, 0.0%–5.6%) of HIV-infected children with radiologically confirmed pneumonia (Figure). Bacteria as a combined group (EF 43.7%; 95% CrI, 31.5%–57.3%) predominated over viruses (EF 26.8%; 95% CrI, 15.7%–38.2%) in their association with under 5 radiologically confirmed pneumonia in HIV-infected children (Figure).

**FIGURE 1.**
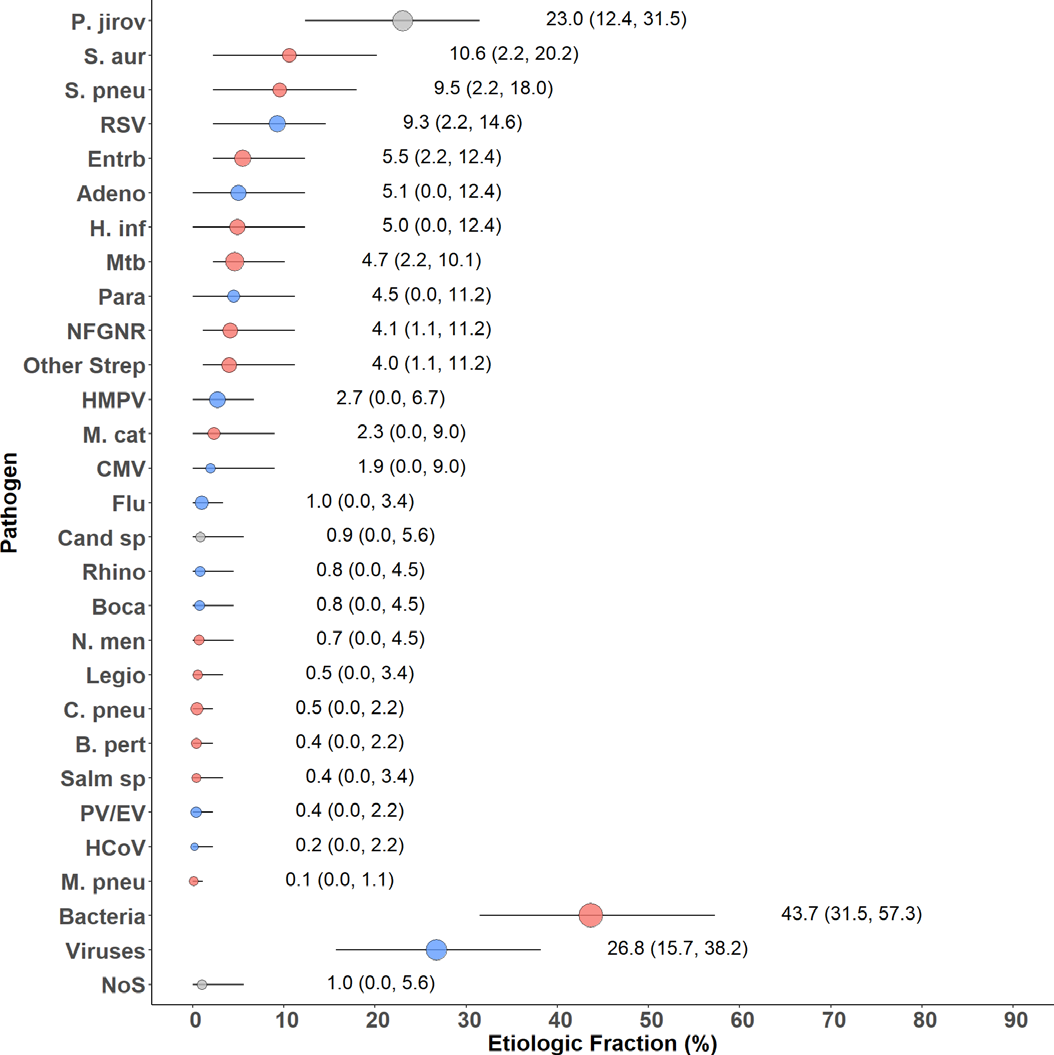
Integrated etiology results for HIV-infected cases with radiologically confirmed pneumonia. Sample size: n = 89. Adeno indicates adenovirus; *B. pert, Bordetella pertussis*; Boca, Human bocavirus; *C. pneu, Chlamydophila pneumoniae*; Cand sp, Candida species; HCoV, human coronavirus; HMPV, human metapneumovirus A/B; *Legio*, Legionella species; *M. cat, Moraxella catarrhalis*; *M. pneu, Mycoplasma pneumoniae*; NFGNR, Nonfermentative Gram-negative rods; *N. men, Neisseria meningitidis*; NoS, not otherwise specified (ie, pathogens not tested for); PV/EV, Parechovirus/Enterovirus; Rhino, human rhinovirus; and Salm sp, Salmonella species. Other Strep includes *Streptococcus pyogenes* and *Enterococcus faecium*. NFGNR includes Acinetobacter species and Pseudomonas species. Enterobacteriaceae includes *E. coli*, Enterobacter species, and Klebsiella species, excluding mixed Gram-negative rods. Radiologically confirmed defined as consolidation and/or other infiltrate on chest radiograph. Bacterial summary excludes *Mtb*. Pathogens estimated at the subspecies level but grouped to the species level for display (Parainfluenza virus type 1, 2, 3 and 4; *S. pneumoniae* PCV13 and *S. pneumoniae* non-PCV13 types; *H. influenzae* type b and *H. influenzae* non-b; influenza A, B, and C). Estimates, including subspecies and serotype disaggregation (eg, PCV13 type and non PCV13 type), are given in Table 3 (age-stratified analysis) and Supplemental Digital Content 7, http://links.lww.com/INF/D849 (pneumonia severity-stratified analysis) for the top 10 pathogens. Description of symbols: Line represents the 95% credible interval. The size of the symbol is scaled based on the ratio of the estimated EF to its standard error. Of 2 identical EF estimates, the estimate associated with a larger symbol is more informed by the data than the priors.

In sensitivity analysis, a lower sensitivity prior (10%–30%) yielded a higher EF (6.0%; 95% CrI, 0.8%–15.7%) of *Mtb* as a contributor to radiologically confirmed pneumonia (data not shown).

### Age- and Pneumonia Severity-stratified Analysis for Etiologic Fraction Estimation

Stratifying the cohort of HIV-infected children by age group emphasized notable differences in the ranking of pathogens associated with radiologically confirmed pneumonia (Supplemental Digital Content 6, http://links.lww.com/INF/D848), although these analyses were compromised by small sample size. *P. jirovecii*, RSV, *Enterobacteriaceae*, nonvaccine-type pneumococcus and *Mtb* contributed a combined EF of 65.9% (95% CrI, 50.0%–79.0%) of radiologically confirmed pneumonia in HIV-infected children <12 months of age. In HIV-infected children 12 to 59 months of age *S. aureus*, PCV13-serotype pneumococcus, nontype b *H. influenzae* and parainfluenza virus (combined EF 62.8%; 95% CrI, 37.0%–85.2%) were the top-ranked pathogens (Supplemental Digital Content 6, http://links.lww.com/INF/D848 and Table 4).

**TABLE 4.** Top 10’ Pathogens Associated With Radiologically Confirmed Pneumonia in HIV-infected Children, Stratified by Age

Age 1–11 months (n = 62)		Age 12–59 months (n = 27)	
Pathogen	EF (95% CrI)	Pathogen	EF (95% CrI)
*P. jirov*	32.6 (17.7–45.2)	*S. aur*	33.7 (7.4–63.0)
RSV	13.0 (1.6–19.4)	*S. pneu* PCV13	13.8 (0.0–37.0)
Entrb	7.5 (3.2–17.7)	*Hi* non-b	9.9 (0.0–25.9)
*S. pneu* non-PCV13	6.5 (1.6–14.5)	Para	5.5 (0.0–14.8)
*Mtb*	6.4 (3.2–14.5)	*M. cat*	4.4 (0.0–22.2)
Adeno	5.6 (0.0–14.5)	CMV	3.8 (0.0–22.2)
NFGNR	5.2 (1.6–14.5)	Adeno	3.8 (0.0–18.5)
Other Strep	5.2 (1.6–14.5)	HMPV	3.2 (0.0–11.1)
Para	4.1 (0.0–12.9)	Flu	2.8 (0.0–11.1)
HMPV	2.5 (0.0–6.5)	NFGNR	1.6 (0.0–11.1)
Top 10	88.5 (74.2–98.4)	Top 10	82.5 (59.3–100)

Adeno indicates adenovirus; CrI, credible interval; CMV, human cytomegalovirus; EF, etiologic fraction; Entrb, Enterobacteriaceae; Flu, influenza virus; Hi non-b, nontype b *Haemophilus influenzae*; HMPV, human metapneumovirus A/B; *M. cat*, *Moraxella catarrhalis*; *Mtb*, *Mycobacterium tuberculosis*; NFGNR, non-fermentative Gram-negative rods; *P. jirov*, *Pneumocystis jirovecii*; Para, parainfluenza virus; PCV13, 13-valent pneumococcal conjugate vaccine; RSV, respiratory syncytial virus A/B; *S. aur*, *Staphylococcus aureus*; *S. pneu* Non-PCV13, non-13-valent PCV type *Streptococcus pneumoniae*; *S. pneu* PCV13, 13-valent PCV type *Streptococcus pneumoniae*.

Other Strep includes *Streptococcus pyogenes* and *Enterococcus faecium*. NFGNR includes Acinetobacter species and Pseudomonas species. Enterobacteriaceae includes *E. coli*, Enterobacter species, and Klebsiella species, excluding mixed Gram-negative rods. Radiologically confirmed defined as consolidation and/or other infiltrate on chest radiograph.

*Mtb* was ranked seventh among pathogens associated with severe (EF 4.2%; 95% CrI, 1.6%–11.5%) and very severe pneumonia (EF 6.6%; 95% CrI, 3.6%–17.9%) (Supplemental Digital Content 7, http://links.lww.com/INF/D849). Viral pathogens, including CMV, contributed a combined EF of 16.8% (95% CrI, 3.3%–36.1%) within the ‘top 10’ pathogens associated with severe pneumonia in HIV-infected children, whereas adenovirus, RSV, parainfluenza virus and human rhinovirus contributed a combined EF of 24.2% (95% CrI, 7.4%–46.4%) within the ‘top 10’ in those with very severe pneumonia (Supplemental Digital Content 7, http://links.lww.com/INF/D849).

## DISCUSSION

This is the first case-control study of pneumonia etiology among HIV-infected children under 5 years of age to have emanated from sub-Saharan Africa in a setting with established access to HibCV and PCV, and widespread availability of ART. Using state-of-the-art molecular diagnostic and statistical analytic techniques, *P. jirovecii* was found to contribute the largest EF (23.0%; 95% CrI, 12.4%–31.5%) of radiologically confirmed pneumonia in HIV-infected children, while *S. aureus* and pneumococcus (ranked second and third in the overall analysis) contributed a combined EF of 20.1%. It is noteworthy that *P. jirovecii* (EF 24.9%; 95% CrI, 15.5%–36.2%), pneumococcus and *S. aureus* were also the top three pathogens associated with radiologically confirmed pneumonia among HIV-infected children in the Zambian PERCH cohort.^38^ At the Zambia site, however, pneumococcus contributed a much larger EF (19.8%; 95% CrI, 8.6%–36.2%) to overall radiologically confirmed pneumonia in HIV-infected children.^38^

The assertion that widespread vaccine coverage could shift the etiologic spectrum of pneumonia in settings where Hib and pneumococcus were once the predominant causes of pneumonia hospitalization in low-middle income settings, is one of the key motivations for which PERCH was conducted.^39^ Bacteria contributed a combined EF of 43.7% to the burden of hospitalized radiologically confirmed pneumonia in South African HIV-infected children and 50.4% in Zambia.^38^ In contrast, the contribution of viruses to the EF of pneumonia in South African HIV-infected children (EF 26.8%) was greater than that observed in the HIV-infected Zambian PERCH cases (EF 17.1%).^38^ Taken together, the bacterial-viral ratio to overall pneumonia etiology was 1.6:1 (43.7/26.8) in South Africa and 2.9:1 (50.4/17.1) in Zambia, with the most striking difference in bacterial etiology between the sites being a much higher burden of pneumococcal pneumonia in Zambia.

Although numerous extrinsic factors likely impact on under 5 pneumonia etiology profiles between the South African and Zambian PERCH cohorts, 2 major contributors to differences observed between the HIV-infected etiology analyses deserve mention here. First, while South Africa introduced PCV into its national immunization program in 2009 and coverage was similar in HIV-infected cases and controls (67.9% and 62.6%, respectively), Zambia introduced PCV in the last 3 months of PERCH enrollment activities and therefore represented a PCV-unvaccinated context.^38^ Second, there was wider ART coverage among South African HIV-infected children enrolled in PERCH (30.0% cases and 83.1% controls) compared with Zambian HIV-infected children (13.6% cases and 41.2% controls), despite similar national estimates for ART coverage (45% in South Africa and 54% in Zambia).^22,38^ Using a vaccine-probe approach, pneumococcus was estimated to have caused 15% (95% CI, 5%–24%) of clinical pneumonia episodes requiring hospitalization in HIV-infected South African children in the pre-PCV, pre-ART era.^40^ The impact of PCV immunization in South Africa, which has led to reductions in invasive pneumococcal disease, including bacteremic pneumococcal pneumonia,^41^ and all-cause pneumonia^42^ may have contributed to the lower pneumococcal EF (9.5%; 95% CrI, 2.2%–18.0%) in HIV-infected South African children in PERCH.

*S. aureus* contributed a similar EF to overall radiologically confirmed pneumonia in South Africa (10.6%; 95% CrI, 2.2%–20.2%) and Zambia (12.7%; 95% CrI, 0.0%–25.9%).^38^ In age-stratified analyses of the HIV-infected cases, *S. aureus* was associated with radiologically confirmed pneumonia among South African children ≥12 months of age (EF 33.7%; 95% CrI, 7.4%–63.0%) but to a lesser extent in Zambian children ≥12 months of age (EF 0.6%; 95% CrI, 0.0%–5.9%).^38^ Conversely, *S. aureus* contributed to disease in Zambian infants (EF 17.8%; 95% CrI, 0.0%–36.6%)^38^ but to a lesser extent in South African infants (0.5%; 95% CrI, 0.0%–4.8%). Differences in the epidemiology of nasopharyngeal carriage of *S. aureus* may relate to climatic/seasonal, socioeconomic, genetic or other factors,^43^ which could potentially impact on the epidemiology of *S. aureus* associated pneumonia in South Africa and Zambia. A well-characterized inverse relationship between *S. aureus* and pneumococcal nasopharyngeal carriage has been noted in South African and Gambian pediatric cohorts with widespread PCV coverage.^44,45^ Prevalence of pneumococcal and staphylococcal nasopharyngeal carriage in HIV-infected PERCH cases in South Africa (65.2% and 45.2%) and Zambia (78.5% and 24.7%)^38^ suggest that widespread PCV coverage may promote higher nasopharyngeal *S. aureus* colonization rates. Although an increasing burden of staphylococcal pneumonia has not been definitively described in the era of access to PCV, ongoing surveillance to detect a shift from pneumococcal to *S. aureus* associated pneumonia must be undertaken in communities with high PCV coverage.^46^

Respiratory viruses featured less prominently in South African HIV-infected children (EF 26.8%; 95% CrI, 15.7%–38.2%) than they did among those that were HIV-uninfected (EF 54.7%; 95% CrI, 47.4%–62.0%).^10^ RSV (EF 9.3%; 95% CrI, 2.2%–14.6%) featured as the most important viral pathogen in South African HIV-infected children, as was found in HIV-uninfected children^10^ but clustered almost exclusively among infants (EF 13.0%; 95% CrI, 1.6%–19.4%). CMV, an important opportunistic pathogen in other HIV-infected pediatric pneumonia etiology studies,^47^ did not feature as being an important contributor to the burden of disease in our analyses, contributing only 1.9% (95% CrI, 0.0%–9.0%) to the overall EF in South African HIV-infected children.

*P. jirovecii*, although the top-ranked pathogen associated with radiologically confirmed pneumonia in South Africa and Zambia,^38^ featured exclusively among children <12 months of age at both sites. In the pre-ART era, *P. jirovecii* was identified (usually through using clinical parameters and/or immunofluorescent staining techniques) in 10% to 35% of South African HIV-infected infants hospitalized with acute pneumonia.^6,8,9^ Given the lower sensitivity of immunofluorescence compared with PCR in the diagnosis of *P. jirovecii* pneumonia in infants,^48^ some of these early studies from South Africa likely underestimated the burden of pneumonia associated with *P. jirovecii*. Nevertheless, such a high proportion of disease attributable to an opportunistic pathogen in HIV-infected children, despite widespread access to ART, is concerning and speaks to gaps in prevention of mother-to-child transmission (PMTCT) coverage in communities with a high burden of maternal antenatal HIV seroprevalence. Since 2015, 3 years after PERCH enrollment activities ended at the South African site, South African PMTCT guidelines emphasize the importance of testing mothers for their HIV serostatus using rapid tests 3-monthly throughout pregnancy, at delivery, at the 6-week immunization visit and 3-monthly during breast-feeding.^49^ Routine birth PCR testing among HIV-exposed neonates and expedited initiation of ART for those confirmed to be HIV-infected, was piloted in late 2013 and has since become standard-of-care in South Africa.^49^ It is anticipated that access to ART in the youngest HIV-infected infants will reduce the burden of *P. jirovecii* associated pneumonia in our setting.

*Mtb* contributed 4.7% (95% CrI, 2.2%–10.1%) of radiologically confirmed pneumonia in the South African HIV-infected cohort, which was lower than the EF of *Mtb* in HIV-uninfected children at the South African site (11.6% and 8.3% in HIV-exposed and HIV-unexposed children, respectively).^10^ Among HIV-infected children with radiologically confirmed pneumonia in Zambia, *Mtb* contributed a similar EF (4.5%) of disease.^38^ The higher sensitivity prior (20%–50%) chosen for *Mtb* in the current analysis likely gave rise to minimal estimates of the burden of *Mtb* in HIV-infected children hospitalized with WHO severe/very severe pneumonia. Eleven percent of South African HIV-infected cases had evidence of *Mtb* infection on tuberculin skin testing. These findings emphasize the importance of performing a diagnostic work-up for *Mtb*, including submission of specimens for *Mtb* culture, in young children hospitalized with radiologically confirmed pneumonia in sub-Saharan Africa.

This study presents novel insights into the etiology of WHO-defined severe/very severe pneumonia among HIV-infected children under 5 years of age in a setting with a mature ART program, effective PMTCT and access to bacterial conjugate vaccines. Study strengths include the rigor with which clinical and laboratory procedures were standardized for ease of comparison between sites and for wider application to other geographic contexts in the developing world. An important limitation of this analysis is the relatively small sample size of HIV-infected cases enrolled into the study, a consequence of which are point estimates with wide CrIs in our estimates of presumptive etiology among radiologically confirmed pneumonia cases. Lower numbers of HIV-infected cases enrolled in South Africa into PERCH (n = 115) compared with the sample size anticipated during the planning stages of the study (n = 400) bears testament to the success of PMTCT and ART programs in limiting vertical transmission of HIV from mother-to-child and preventing hospitalization in HIV-infected children accessing ART. Wide CrIs of EFs, particularly in stratified analyses, emanate from the small number of children in the cohort.

## CONCLUSIONS

*P. jirovecii* is the major contributor to radiologically confirmed pneumonia in HIV-infected children under 5 years of age in South Africa but almost exclusively in children <12 months of age. This highlights the need for intensified efforts to expedite HIV diagnosis and initiation onto ART in South Africa and other high-burdened settings of endemic HIV seroprevalence. In children ≥12 months of age, the dominant pathogens were *S. aureus*, pneumococcus and *H. influenzae*. These combined results suggest that the initial therapy for HIV-infected South African children presenting with CAP should include empiric treatment with antibiotics targeting these pathogens.

## ACKNOWLEDGMENTS

The authors are grateful for the participation of all of the children and their families who participated in PERCH at the South African site. Substantial input with regards site-specific study and laboratory set-up were made by Michelle J. Groome and Peter V. Adrian at the Respiratory & Meningeal Pathogens Research Unit at Chris Hani Baragwanath Academic Hospital. Substantial oversight of PERCH activities was made by Amanda J. Driscoll through the Department of International Health, International Vaccine Access Center, Johns Hopkins Bloomberg School of Public Health, Baltimore, MD. Socio-economic stratification of PERCH participants was derived through analyses that were conducted by Elizabeth Chmielewski-Yee. Data quality assurance was provided by Nora L. Watson at The Emmes Corporation, Rockville, MD, and the Bayesian analysis was undertaken by Zhenke Wu and Scott L. Zeger at the Department of Biostatistics, Johns Hopkins University, Baltimore, MD. We acknowledge members of the PERCH Chest Radiograph Reading Panel and Shalika Jayawardena and Rose Watt from Canterbury Health Laboratories. We also acknowledge the substantial contributions of the other members of the PERCH Study Group: Johns Hopkins Bloomberg School of Public Health, Baltimore, MD: Orin S. Levine (former principal investigator; current affiliation: Bill & Melinda Gates Foundation, Seattle, WA), Andrea N. DeLuca, Nicholas Fancourt, Wei Fu, E. Wangeci Kagucia, Ruth A. Karron, Mengying Li, Daniel E. Park, Qiyuan Shi; Department of Clinical Medicine, University of Oxford, United Kingdom: Jane Crawley; Medical Research Council, Basse, The Gambia: Stephen R. C. Howie (site principal investigator); KEMRI-Wellcome Trust Research Programme, Kilifi, Kenya: J. Anthony G. Scott (site principal investigator and PERCH co-principal investigator, joint affiliation with London School of Hygiene and Tropical Medicine, London, UK); Division of Infectious Disease and Tropical Pediatrics, Department of Pediatrics, Center for Vaccine Development, University of Maryland School of Medicine, Baltimore, MD and Centre pour le Développement des Vaccins (CVD-Mali), Bamako Mali: Karen L. Kotloff (site principal investigator); International Centre for Diarrhoeal Disease Research, Bangladesh (icddr,b): W. Abdullah Brooks (site principal investigator); Thailand Ministry of Public Health – U.S. CDC Collaboration, Nonthaburi, Thailand: Henry C. Baggett (site principal investigator), Susan A. Maloney former site principal investigator); Boston University School of Public Health, Boston, MA, and University Teaching Hospital, Lusaka, Zambia: Donald M. Thea (site principal investigator); Canterbury Health Laboratories, Christchurch, New Zealand: Trevor P. Anderson, Joanne Mitchell.

## Supplementary Material


