# Supplementary material for: The Etiology of Pneumonia in HIV-1-infected South African Children in the Era of Antiretroviral Treatment: Findings From the Pneumonia Etiology Research for Child Health (PERCH) Study
Source: Pediatr Infect Dis J. 2021 Aug 25;40(9):S69–78. doi: 10.1097/INF.0000000000002651 (PMC8448402; doi:10.1097/INF.0000000000002651)
Supplement: Supplementary file 1 [file inf-40-s69-s001.docx]

# SUPPLEMENTAL DIGITAL CONTENT 1

Information contained in Supplemental Digital Content 1 details aspects of human immunodeficiency type-1 (HIV)-infected pediatric care at the South African Pneumonia Etiology Research for Child Health (PERCH) study site, situated at Chris Hani Baragwanath Academic Hospital (CHBAH) in Soweto, Gauteng Province. It also highlights the inherent biases embedded in our approach to HIV-infected control enrolment.

## Nationwide Care of HIV-infected Children

The South African national antiretroviral therapy (ART) roll-out commenced for public sector patients in 2004, with gradually improving coverage for adults and children in need of ART over time.^(1, 2)^

The standard of care for HIV-infected children <1 year of age in South Africa at commencement of the PERCH study was to initiate ART immediately upon diagnosis irrespective of the CD4+ T-helper cell count, a strategy which had been earlier shown to improve survival in HIV-infected infants.^(3)^ In 2011 through end-August 2012, children between the ages of one and five years were eligible for initiation onto ART if diagnosed as having World Health Organization (WHO) Stage IV disease, or if CD4+ T-helper cell counts were <750 cells/μL.^(4)^ Subsequent to August 2012, any HIV-infected child <5 years of age was deemed eligible for initiation onto ART, regardless of the degree of immunologic suppression.^(5)^ First-line ART regimens consisted of abacavir and lamivudine with either lopinavir/ritonavir (for children <3 years of age and <10 kilograms in weight), or efavirenz (for children ≥3 years of age and weighing ≥10 kilograms) according to national guidelines.^(6, 7)^ In 2010/2011, 19% of children hospitalized in the general pediatric wards at CHBAH were HIV-infected.^(8)^

## HIV-infected Control Selection Procedure

It would have been difficult to adopt a community-based screening strategy for the purposes of HIV-infected control enrolment at the South African PERCH site, because of the low population prevalence (≤2%) of HIV infection in children <5 years of age in the study catchment area.^(9)^ We therefore used a convenience sample of healthy children attending outpatient ART clinics at CHBAH as our HIV-infected control group, and designate these as being ART-clinic controls.

There are two outpatient ART clinics for the follow-up of HIV-infected children at CHBAH. Harriet Shezi Children’s HIV Clinic is run as part of the public health sector services, and follows the care of HIV-infected children previously diagnosed in the general pediatric wards at CHBAH. The clinic also assists with the care of children failing ART at Soweto community health clinics. The Perinatal HIV Research Unit (PHRU) Clinic is a research-focussed entity which manages HIV-infected infants and children enrolled in clinical trials.

Virtually all ART Clinic attendees were on ART and receiving co-trimoxazole prophylaxis at the time of enrolment into PERCH. This would necessarily impose bias in our HIV-infected control group, which would be expected to have been relatively protected against developing community-acquired pneumonia because of the beneficial effects of ART and routine use of co-trimoxazole prophylaxis.

## Specimen Collection from HIV-infected Cases and Controls

Cases and controls underwent collection of a flocked nasopharyngeal (NP) swab (flexible minitip, Copan®, Murrieta, CA), which was pooled with a rayon oropharyngeal (OP) swab (Fisher Scientific®, Loughborough, UK) in 3 mL universal transport medium (Copan®, Murrieta, CA) for multiplex respiratory pathogen polymerase chain reaction (PCR) testing (Fast Track Diagnostics Respiratory Pathogens 33 test, Fast Track Diagnostics, Sliema, Malta). A second NP specimen, using a rayon swab placed into 1 mL skim milk, tryptone, glucose, glycerol (STGG) transport medium was collected for pneumococcal culture and serotyping.^(10-12)^

Additionally, cases and ART-clinic controls had blood samples collected for *lytA* PCR testing (for detection of pneumococcal DNAemia).^(13)^ ART-clinic controls had EDTA blood submitted for hemoglobin testing, and a subset had their blood tested for C-reactive protein.

HIV infection status was confirmed in children <18 months of age using qualitative HIV PCR (Roche COBAS® AmpliScreen HIV-1 Test, version 1.5), and children ≥18 months of age were tested using enzyme-linked immunosorbent assay (ELISA). CD4+ T-helper cell counts were determined on a FC500 MPL analyser (Beckman-Coulter, Indianapolis, IN) using the single platform panleucogated method.^(14)^ CD4+ T-helper cell count determinations collected as part of routine clinical care within 30 days pre- or post-enrollment into PERCH were considered for analysis if these assays were not performed on the day of enrollment (ART-clinic controls) or during the course of the PERCH hospitalization episode (cases). Immunological stage of HIV infection was attributed using the WHO staging system.^(15)^

Serum specimens were collected from cases and ART-clinic controls, for determination of antibiotic activity at the time of enrollment into PERCH.

## The PERCH Integrated Analysis

The percent of pneumonia due to each pathogen was estimated using the PERCH Integrated Analysis (PIA) method, which is described in detail elsewhere.^(16-18)^ In brief, the PIA is a Bayesian nested partially latent class analysis that integrates the results for each case from blood culture, NP/OP PCR, whole blood PCR for pneumococcus and induced sputum culture for *Mycobacterium tuberculosis* (*Mtb*). The PIA also integrates test results from controls to account for imperfect test specificity of NP/OP PCR and whole blood PCR. Blood culture results (excluding contaminants) and *Mtb* results were assumed to be 100% specific.

The PIA accounts for imperfect sensitivity of each test/pathogen measurement by using *a priori* estimates of their sensitivity (i.e., estimates regarding the plausibility range of sensitivity which varied by laboratory test method and pathogen). Sensitivity of blood culture was reduced if blood volume was low (<1.5 mL) or if antibiotics were administered before specimen collection. Sensitivity of NP/OP PCR for *Streptococcus pneumoniae* and *Haemophilus influenzae* was reduced if antibiotics were administered before specimen collection (Table).

As a Bayesian analysis, both the list of pathogens and their starting ‘prior’ etiologic fraction values were specified *a priori,* which favored no pathogen over another (i.e., ‘uniform’). The pathogens selected for inclusion in the analysis included any non-contaminant bacteria detected by culture in blood at any of the 9 PERCH sites, regardless of whether it was observed at the South African site specifically, *Mtb*, and all of the multiplex quantitative PCR pathogens except those considered invalid because of poor assay specificity (*Klebsiella* *pneumoniae*^(19)^ and *Moraxella catarrhalis*). A category called ‘Pathogens Not Otherwise Specified’ (NoS) was also included to estimate the fraction of pneumonia caused by pathogens not tested for or not observed. A child negative for all pathogens would still be assigned an etiology, which would be either one of the explicitly estimated pathogens (implying a ‘false negative’, accounting for imperfect sensitivity of certain measurements) or NoS.

All analyses were adjusted for age (<1 vs ≥1 year) to account for differences in pathogen prevalence among controls. For results stratified by case clinical data (e.g., to radiologically-confirmed pneumonia, very severe, etc.), the test results from all controls were used. However, for analyses stratified by age, only data from controls representative of that age group were used. The model assessing etiology by severity was not adjusted for age due to limited sample size.

The PIA estimated both the individual and population-level etiology probability distributions, each summing to 100% across pathogens where each pathogen has a probability ranging from 0% to 100%. The population level etiologic fraction estimate for each pathogen was approximately the average of the individual case probabilities and was provided with a 95% Credible Interval (95% CrI), the Bayesian analogue of the confidence interval.

**Table: Integrated etiology analysis input values for sensitivity and specificity of laboratory test measures**

|  |  | **Sensitivity Prior^a^** | |  |
| --- | --- | --- | --- | --- |
| **Specimen/test** | **Pathogen** | **Base^b^** | **Reduced^b^** | **Specificity** |
| Blood cultures^c^ | *Streptococcus pneumoniae*  *Haemophilus influenzae* | 5-20% | 1-13% | 100% |
|  | *Moraxella catarrhalis*  *Staphylococcus aureus*  Non-fermentative Gram-negative rods  Candida species  Non-pneumococcal streptococci, including enterococci | 5-15% | 1-10% |  |
|  | Salmonella species  Enterobacteriaceae  *Neisseria meningitidis* | 10-50% | 1-34% |  |
| NP/OP PCR | *Streptococcus pneumoniae*  *Haemophilus influenzae* | 50-90% | 15-55% | 1 − Control prevalence (ref Table 2) |
|  | Salmonella species  Legionella species | 0.5-90% | 0.5-90% |  |
|  | All other PCR targets | 50-90% | 50-90% |  |
| Whole blood PCR | *Streptococcus pneumoniae* | 12-65% | 12-65% | 1 − Control prevalence (ref Table 2) |
| Induced sputum | *Mycobacterium tuberculosis* | 20-50% | 20-50% | 100% |

a. Background information supporting choice of sensitivity priors provided in the all-site PERCH paper.^(18)^

b. Base: >1.5 mL blood culture volume (blood culture only) and no evidence of prior antibiotic exposure. Reduced <1.5 mL, or evidence of prior antibiotic exposure.

c. Direct evidence of the diagnostic sensitivity for *Streptococcus pneumoniae* and *Haemophilus influenzae* from vaccine probe studies. For all other pathogens, we set the base blood culture sensitivity prior to 5-15%, with the exception of Salmonella species, Enterobacteriaceae and *Neisseria meningitidis*, for which we selected wider priors (10-50%) to reflect their greater uncertainty.

## References

1. Nattrass N. South Africa's "rollout" of highly active antiretroviral therapy: a critical assessment. *J Acquir Immune Defic Syndr*. 2006;43:618-623.

2. Nunes MC, von Gottberg A, de Gouveia L, et al. The impact of antiretroviral treatment on the burden of invasive pneumococcal disease in South African children: a time series analysis. *AIDS*. 2011;25:453-462.

3. Violari A, Cotton MF, Gibb DM, et al. Early antiretroviral therapy and mortality among HIV-infected infants. *N Engl J Med*. 2008;359:2233-2244.

4. World Health Organization. *Antiretroviral therapy for HIV infection in infants and children: towards universal access*. Geneva: World Health Organization; 2010.

5. World Health Organization. *Consolidated Guidelines on the use of Antiretroviral Drugs for Treating and Preventing HIV Infection*. Geneva: World Health Organization,; 2013.

6. South African National Department of Health. Guidelines for the Management of HIV in Children. In: South African Department of Health, ed. Second ed. Pretoria: South African National Department of Health; 2010.

7. South African National Department of Health. Guidelines for the Management of Tuberculosis in Children. In: South African National Department of Health, ed. Pretoria: South African National Department of Health; 2013.

8. Meyers T, Dramowski A, Schneider H, Gardiner N, Kuhn L, Moore D. Changes in paediatric HIV-related hospital admissions and mortality in Soweto, South Africa 1996-2011: light at the end of the tunnel? *J Acquir Immune Defic Syndr*. 2012;60:503-10.

9. Actuarial Society of South Africa (ASSA). AIDS and Demographic Model 2008. Available at: <https://www.actuarialsociety.org.za/download/assa-aids-model-2008-full/>. Accessed 30 January, 2017.

10. Baggett HC, Watson NL, Deloria Knoll M, et al. Density of Upper Respiratory Colonization With *Streptococcus pneumoniae* and Its Role in the Diagnosis of Pneumococcal Pneumonia Among Children Aged <5 Years in the PERCH Study. *Clin Infect Dis*. 2017;64:S317-s327.

11. Feikin DR, Fu W, Park DE, et al. Is Higher Viral Load in the Upper Respiratory Tract Associated With Severe Pneumonia? Findings From the PERCH Study. *Clin Infect Dis*. 2017;64:S337-s346.

12. Park DE, Baggett HC, Howie SRC, et al. Colonization Density of the Upper Respiratory Tract as a Predictor of Pneumonia - *Haemophilus influenzae*, *Moraxella catarrhalis*, *Staphylococcus aureus*, and *Pneumocystis jirovecii*. *Clin Infect Dis*. 2017;64:S328-s336.

13. Deloria Knoll M, Morpeth SC, Scott JAG, et al. Evaluation of Pneumococcal Load in Blood by Polymerase Chain Reaction for the Diagnosis of Pneumococcal Pneumonia in Young Children in the PERCH Study. *Clin Infect Dis*. 2017;64:S357-s367.

14. Glencross DK, Janossy G, Coetzee LM, et al. Large-scale affordable PanLeucogated CD4+ testing with proactive internal and external quality assessment: in support of the South African national comprehensive care, treatment and management programme for HIV and AIDS. *Cytometry B Clin Cytom*. 2008;74 Suppl 1:S40-51.

15. World Health Organization. WHO Case Definitions of HIV for Surveillance and Revised Clinical Staging and Immunological Classification of HIV-related Disease in Adults and Children. In: World Health Organization, ed. Geneva: World Health Organization.

16. Wu Z, Deloria-Knoll M, Zeger SL. Nested partially latent class models for dependent binary data; estimating disease etiology. *Biostatistics*. 2016;18:200-213.

17. Deloria Knoll M, Fu W, Shi Q, et al. Bayesian Estimation of Pneumonia Etiology: Epidemiologic Considerations and Applications to the Pneumonia Etiology Research for Child Health Study. *Clin Infect Dis*. 2017;64:S213-s227.

18. Pneumonia Etiology Research for Child Health (PERCH) Study Group. Causes of severe pneumonia requiring hospital admission in children without HIV infection from Africa and Asia: the PERCH multi-country case-control study. *Lancet*. 2019;394:757-779.

19. Zar HJ, Barnett W, Stadler A, Gardner-Lubbe S, Myer L, Nicol MP. Aetiology of childhood pneumonia in a well vaccinated South African birth cohort: a nested case-control study of the Drakenstein Child Health Study. *Lancet Respir Med*. 2016;4:463-472.
