# Supplementary material for: The Etiology of Pneumonia in HIV-1-infected South African Children in the Era of Antiretroviral Treatment: Findings From the Pneumonia Etiology Research for Child Health (PERCH) Study
Source: Pediatr Infect Dis J. 2021 Aug 25;40(9):S69–78. doi: 10.1097/INF.0000000000002651 (PMC8448402; doi:10.1097/INF.0000000000002651)
Supplement: Supplementary file 3 [file inf-40-s69-s003.docx]

## Supplemental Digital Content 3: Demographic and Clinical Characteristics of HIV-infected Cases and ART-Clinic Controls Enrolled into PERCH at the South African Site (Additional Data)

| Characteristic | All Cases (n=115) | CXR+ Cases (n=89) | ART-Clinic Controls (n=136) | OR (95% CI);  Adjusted P-value ^a^ | |
| --- | --- | --- | --- | --- | --- |
|  |  |  |  | All cases compared to ART-Clinic Controls | CXR+ cases compared to ART-Clinic Controls |
| Age Category | | | | | |
| 1-5 months | 49/115 (42.6) | 35/89 (39.3) | 45/136 (33.1) | Ref | Ref |
| 6-11 months | 34/115 (29.6) | 27/89 (30.3) | 40/136 (29.4) | 0.76 (0.41, 1.41); 0.544 | 0.84 (0.43, 1.63); 0.824 |
| 12-23 months | 17/115 (14.8) | 16/89 (18.0) | 21/136 (15.4) | 0.75 (0.35, 1.61); 0.631 | 0.98 (0.44, 2.16); 0.986 |
| 24-59 months | 15/115 (13.0) | 11/89 (12.4) | 30/136 (22.1) | 0.46 (0.22, 0.97); 0.093 | 0.47 (0.20, 1.07); 0.150 |
| Season in which Enrolled ^b^ | | | | | |
| Spring | 40/115 (34.8) | 30/89 (33.7) | 43/136 (31.6) | 1.02 (0.51, 2.07); 0.978 | 0.98 (0.46, 2.09); 0.986 |
| Summer | 19/115 (16.5) | 14/89 (15.7) | 16/136 (11.8) | 1.28 (0.54, 3.05); 0.725 | 1.19 (0.47, 3.02); 0.912 |
| Autumn | 32/115 (27.8) | 26/89 (29.2) | 49/136 (36.0) | 0.73 (0.36, 1.49); 0.544 | 0.75 (0.35, 1.61); 0.649 |
| Winter | 24/115 (20.9) | 19/89 (21.3) | 28/136 (20.6) | Ref | Ref |
| Immunization status | | | | | |
| BCG Immunization | 101/105 (96.2) | 76/79 (96.2) | 87/90 (96.7) | 0.90 (0.19, 4.17); 0.977 | 0.87 (0.17, 4.52); 0.966 |
| DTP-Hib Immunization up-to-date ^c^ | 71/107 (66.4) | 52/81 (64.2) | 62/91 (68.1) | 0.93 (0.51, 1.71); 0.958 | 0.86 (0.45, 1.63); 0.833 |
| PCV Immunization up-to-date ^d^ | 72/106 (67.9) | 54/81 (66.7) | 57/91 (62.6) | 1.10 (0.57, 2.14); 0.918 | 1.04 (0.51, 2.12); 0.984 |
| Measles Immunization up-to-date ^e^ | 33/107 (30.8) | 28/81 (34.6) | 36/90 (40.0) | 0.67 (0.30, 1.49); 0.517 | 0.92 (0.40, 2.08); 0.956 |
| Socio-economic status | | | | | |
| Lowest tier | 18/115 (15.7) | 15/89 (16.9) | 20/135 (14.8) | 0.94 (0.36, 2.47); 0.977 | 0.86 (0.32, 2.31); 0.926 |
| Low-to-mid tier | 39/115 (33.9) | 31/89 (34.8) | 34/135 (25.2) | 1.32 (0.56, 3.15); 0.689 | 1.14 (0.47, 2.77); 0.927 |
| Mid-to-upper tier | 43/115 (37.4) | 29/89 (32.6) | 65/135 (48.1) | 0.70 (0.31, 1.59); 0.544 | 0.50 (0.21, 1.18); 0.198 |
| Upper tier | 15/115 (13.0) | 14/89 (15.7) | 16/135 (11.9) | Ref | Ref |
| Anthropometry | | | | | |
| Median WAZ (IQR) | -2.1 (-3.4 to -1.0) | -2.3 (-3.5 to -1.1) | -1.1 (-2.2 to -0.0) | 0.68 (0.57, 0.80); <0.001 | 0.66 (0.54, 0.79); <0.001 |

Abbreviations: ART = Antiretroviral therapy; BCG = Bacillus Calmette-Guérin; CI = Confidence Interval; CXR+ = Radiologically-confirmed pneumonia; DTP = Diphtheria, tetanus, pertussis; Hib = *Haemophilus influenzae* type b; HIV = Human immunodeficiency virus type-1; IQR = Interquartile range; OR = Odds ratio; PERCH = Pneumonia Etiology Research for Child Health study; Ref = Referent; WAZ = Weight-for-age Z-score.

^a^ Odds ratio adjusted by age (in months) and season, and derived by logistic regression analysis. P-values adjusted using the Benjamini-Hochberg method.

^b^ Seasons: Spring (September through November); Summer (December through February); Autumn (March through May); Winter (June through August).

^c^ Complete vaccination defined as receipt of ≥3 doses.

^d^ Complete vaccination defined based on number of doses, and age at first dose, or age at PCV introduction in South Africa: ≥3 doses, or 2 doses if there were at least 8 weeks between doses and the child was <9 months of age at enrolment or >12 months of age at the time of first dose, or ≥1 dose if the age at any of the doses, or age at PCV introduction, was ≥24 months.

^e^ Complete vaccination defined as receipt of at least one dose, restricted to children aged ≥10 months.
