# Supplementary material for: The Etiology of Pneumonia in HIV-1-infected South African Children in the Era of Antiretroviral Treatment: Findings From the Pneumonia Etiology Research for Child Health (PERCH) Study
Source: Pediatr Infect Dis J. 2021 Aug 25;40(9):S69–78. doi: 10.1097/INF.0000000000002651 (PMC8448402; doi:10.1097/INF.0000000000002651)
Supplement: Supplementary file 4 [file inf-40-s69-s004.docx]

## Supplemental Digital Content 4: Clinical Characteristics of HIV-infected Cases, Stratified by Radiologic Findings

| Characteristic | All Cases (n=115) | CXR+ Cases (n=89) |
| --- | --- | --- |
| Very severe pneumonia ^a^ | 39/115 (33.9) | 28/89 (31.5) |
| CXR available | 112/115 (97.4) | 89/89 (100.0) |
| CXR Result | | |
| Any consolidation | 60/112 (53.6) | 60/89 (67.4) |
| Other infiltrate only | 29/112 (25.9) | 29/89 (32.6) |
| Normal | 12/112 (10.7) | - |
| Uninterpretable | 11/112 (9.8) | - |
| Laboratory Results | | |
| Severe anemia ^b^ | 11/114 (9.6) | 7/88 (8.0) |
| Leukocytosis ^c^ | 64/113 (56.6) | 51/87 (58.6) |
| Leukopenia ^d^ | 8/113 (7.1) | 5/87 (5.7) |
| Clinical Parameters | | |
| Hypoxia ^e^ | 90/115 (78.3) | 69/89 (77.5) |
| Fever ≥38°C | 88/115 (76.5) | 66/89 (74.2) |
| Tachycardia | 62/112 (55.4) | 46/87 (52.9) |
| Wheeze on auscultation | 17/113 (15.0) | 13/88 (14.8) |
| Lethargy ^f^ | 11/115 (9.6) | 8/89 (9.0) |
| Duration of illness ^g^ | | |
| Median duration of illness (days, IQR) | 4.0 (2.0-7.0) | 3.5 (2.0-6.5) |
| 0-2 days | 33/114 (28.9) | 26/88 (29.5) |
| 3-5 days | 47/114 (41.2) | 37/88 (42.0) |
| >5 days | 34/114 (29.8) | 25/88 (28.4) |
| Duration of hospitalization | | |
| Median duration of hospitalization (days, IQR) | 8.0 (5.0-16.0) | 10.0 (6.0-17.0) |
| 0-2 days | 10/115 (8.7) | 5/89 (5.6) |
| 3-5 days | 20/115 (17.4) | 14/89 (15.7) |
| >5 days | 85/115 (73.9) | 70/89 (78.7) |
| Died in Hospital | 17/115 (14.8) | 9/89 (10.1) |
| Died post-discharge, within 30 days of admission | 6/115 (5.2) | 6/89 (6.7) |
| Missing 30-day vital status ^h^ | 15/98 (15.3) | 12/80 (15.0) |

Abbreviations: CXR = Chest radiograph; CXR+ = Radiologically-confirmed pneumonia; HIV = Human immunodeficiency virus type-1; IQR = Interquartile range.

^a^ Very severe pneumonia defined as cough or difficulty breathing, and at least one of the following: central cyanosis, difficulty breastfeeding/drinking, vomiting everything, convulsions, lethargy, unconsciousness, or head nodding.

^b^ Severe anemia defined as hemoglobin <7.5 g/dL.

^c^ Defined as leukocyte count >15 × 10^9^ cells/L for children 1-11 months, and >13 × 10^9^ cells/L for children 12-59 months.

^d^ Defined as leukocyte count <5 × 10^9^ cells/L, regardless of age category.

^e^ Hypoxemia defined as room air oxygen saturation <90%, or on supplemental oxygen if a room air oxygen saturation reading was not available.

^f^ Lethargic or unresponsive (responds to voice or pain, unresponsive, or pharmacologically sedated).

^g^ Duration of illness defined as duration (in days) of cough, wheeze, fever, or difficulty breathing, whichever is longest.

^h^ Restricted to those children discharged alive.
