# Supplementary material for: The Etiology of Pneumonia in HIV-1-infected South African Children in the Era of Antiretroviral Treatment: Findings From the Pneumonia Etiology Research for Child Health (PERCH) Study
Source: Pediatr Infect Dis J. 2021 Aug 25;40(9):S69–78. doi: 10.1097/INF.0000000000002651 (PMC8448402; doi:10.1097/INF.0000000000002651)
Supplement: Supplementary file 5 [file inf-40-s69-s005.docx]

## Supplemental Digital Content 5: Microbiology and Mantoux Results in HIV-infected Cases

|  | All cases (n=115) | CXR+ Cases (n=89) | Died in Hospital (n=17) |
| --- | --- | --- | --- |
| Blood Cultures | | | |
| Total blood cultures | 115/115 (100.0) | 89/89 (100.0) | 17/17 (100.0) |
| Contaminants | 15/115 (13.0) | 13/89 (14.6) | 5/17 (29.4) |
| Significant isolates | 9/115 (7.8) | 6/89 (6.7) | 3/17 (17.6) |
| Gram negatives | 6/9 (66.7) | 3/6 (50.0) | 3/3 (100.0) |
| *E. cloacae* | 1 | 1 | 1 |
| *E. coli* | 1 | 1 | 0 |
| *P. aeruginosa* | 3 | 1 | 2 |
| Salmonella spp. | 1 | 0 | 0 |
| Gram positives | 3/9 (33.3) | 3/6 (50.0) | 0/3 (0.0) |
| *E. faecium* | 1 | 1 | 0 |
| *S. pneumoniae* | 1 | 1 | 0 |
| *S. aureus* | 1 | 1 | 0 |
| Flag positive, blood culture negative | | | |
| Pneumococcal latex positive | 1 | 1 | 0 |
| Lung Aspirates | | | |
| Lung aspirate PJP microscopy | 1/1 (100.0) | 1/1 (100.0) | - |
| Pos PJP Immunofluorescence | 0/1 (0.0) | 0/1 (0.0) | - |
| Lung aspirate cultures |  |  |  |
| No organism detected | 1 | 1 | 0 |
| Lung aspirate PCR | | | |
| *S. pneumoniae* | 1 | 1 | 0 |
| Induced Sputum Specimens (IS) | | | |
| Number of children with IS Specimens | 100/115 (87.0) | 80/89 (89.9) | 11/17 (64.7) |
| IS PJP microscopy | 68/100 (68.0) | 57/80 (71.2) | 9/11 (81.8) |
| Pos PJP Immunofluorescence | 1/68 (1.5) | 1/57 (1.8) | 0/9 (0.0) |
| IS *Mtb* Culture Pos | 0/100 (0.0) | 0/80 (0.0) | 0/11 (0.0) |
| Gastric Aspirate Samples (GA) | | | |
| Number of children with GA Specimens | 80/115 (69.6) | 64/89 (71.9) | 7/17 (41.2) |
| GA *Mtb* Culture Pos | 2/80 (2.5) | 2/64 (3.1) | 0/7 (0.0) |
| Endotracheal Tube Specimens (ETT) | | | |
| Number of children with ETT Specimens | 7/115 (6.1) | 6/89 (6.7) | 1/17 (5.9) |
| ETT PJP microscopy | 3/7 (42.9) | 3/6 (50.0) | 0/1 (0.0) |
| Pos PJP Immunofluorescence | 1/3 (33.3) | 1/3 (33.3) | - |
| ETT *Mtb* Culture Pos | 1/7 (14.3) | 1/6 (16.7) | 0/1 (0.0) |
| Tuberculin Skin Tests | | | |
| Total Mantoux tests | 65/115 (56.5) | 53/89 (59.6) | 8/17 (47.1) |
| Neg | 58/65 (89.2) | 48/53 (90.6) | 8/8 (100.0) |
| Pos | 7/65 (10.8) | 5/53 (9.4) | 0/8 (0.0) |

Abbreviations: CXR+ = Radiologically-confirmed pneumonia; ETT = Endotracheal tube; HIV = Human immunodeficiency virus type-1; GA = Gastric aspirate; IS = Induced sputum; *Mtb* = *Mycobacterium tuberculosis*; Neg = Negative; PJP = *Pneumocystis jirovecii*; Pos = Positive.
