# Supplementary figures and images for: The Etiology of Pneumonia in HIV-1-infected South African Children in the Era of Antiretroviral Treatment: Findings From the Pneumonia Etiology Research for Child Health (PERCH) Study
Source: Pediatr Infect Dis J. 2021 Aug 25;40(9):S69–78. doi: 10.1097/INF.0000000000002651 (PMC8448402; doi:10.1097/INF.0000000000002651)

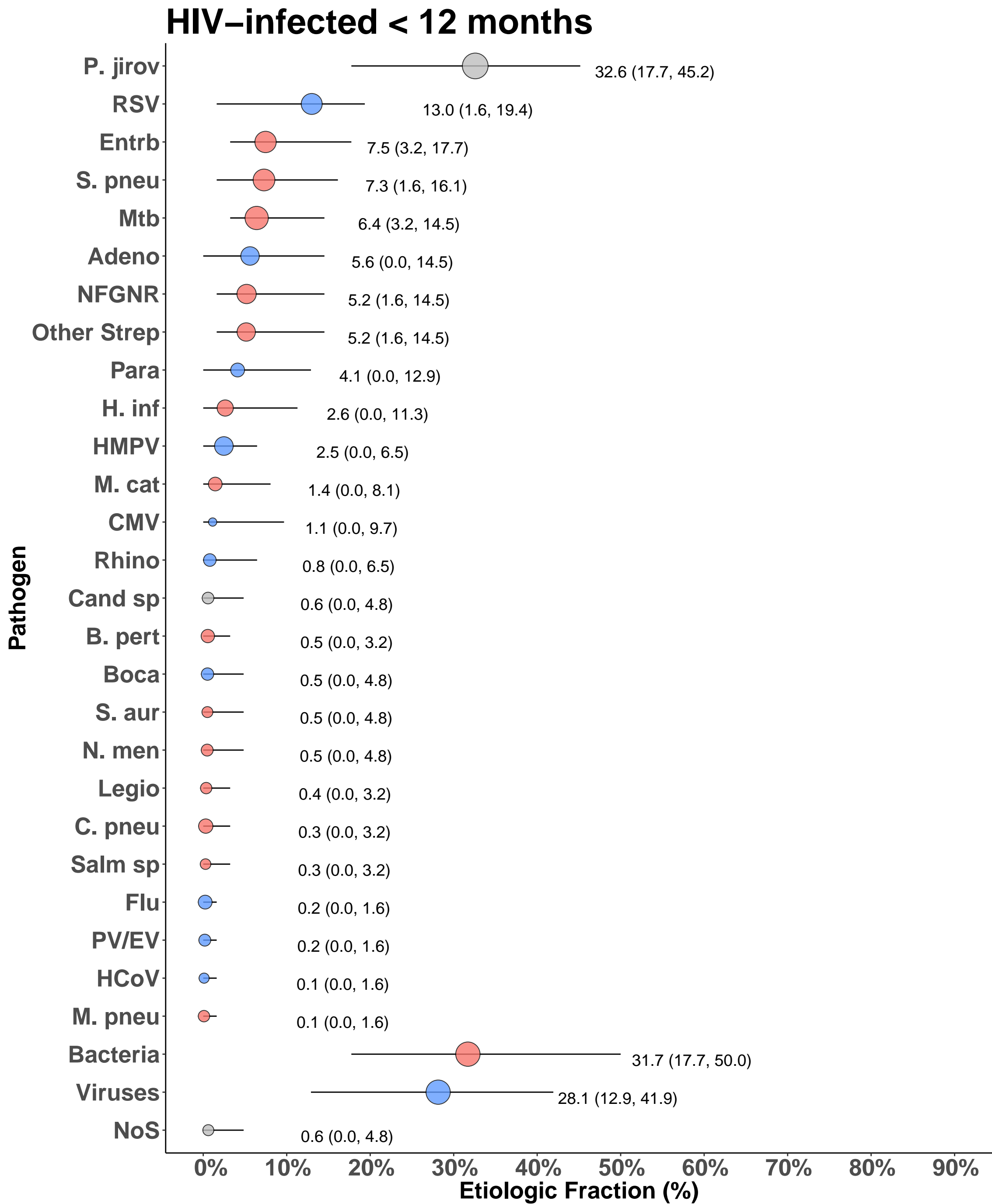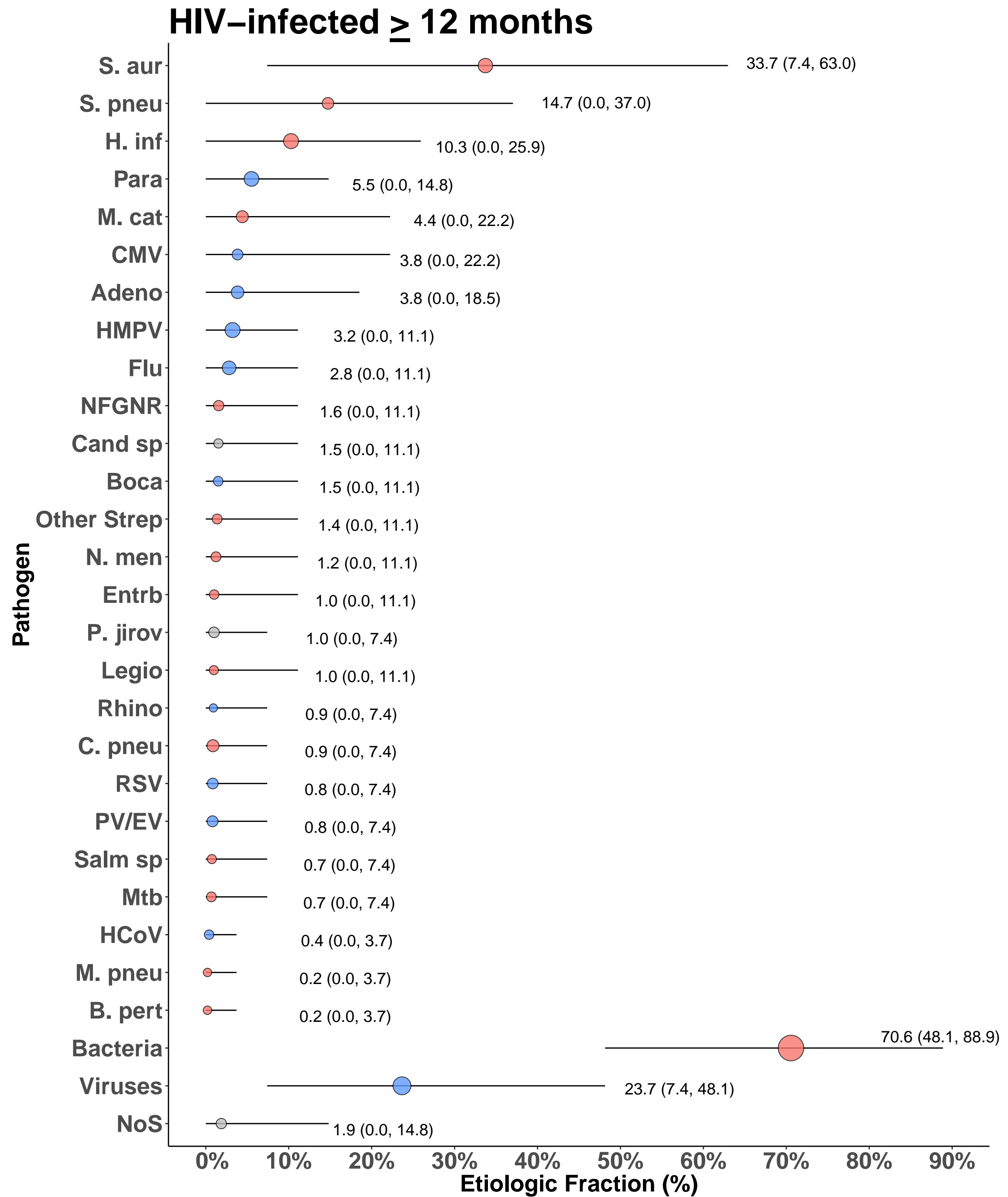

Supplement: Supplementary file 6 [file inf-40-s69-s006.pdf]
