# Supplementary material for: The Etiology of Pneumonia in HIV-1-infected South African Children in the Era of Antiretroviral Treatment: Findings From the Pneumonia Etiology Research for Child Health (PERCH) Study
Source: Pediatr Infect Dis J. 2021 Aug 25;40(9):S69–78. doi: 10.1097/INF.0000000000002651 (PMC8448402; doi:10.1097/INF.0000000000002651)
Supplement: Supplementary file 7 [file inf-40-s69-s007.docx]

## Supplemental Digital Content 7: ‘Top 10’ Pathogens Associated with Radiologically-confirmed Pneumonia in HIV-infected Children, Stratified by Pneumonia Severity

| Severe Pneumonia (n=61) | | Very Severe Pneumonia (n=28) | |
| --- | --- | --- | --- |
| Pathogen | EF (95% CrI) | Pathogen | EF (95% CrI) |
| *P. jirov* | 21.8 (0.0, 37.7) | *S. pneu* Non-PCV13 | 16.2 (3.6, 28.6) |
| *S. pneu* PCV13 | 15.1 (3.3, 26.2) | Adeno | 13.6 (0.0, 32.1) |
| *S. aur* | 11.9 (1.6, 29.5) | Entrb | 12.7 (7.1, 25.0) |
| *Hi* non-b | 8.4 (0.0, 21.3) | *P. jirov* | 9.7 (0.0, 25.0) |
| CMV | 5.5 (0.0, 24.6) | NFGNR | 8.8 (3.6, 25.0) |
| HMPV | 5.2 (0.0, 11.5) | Other Strep | 8.6 (3.6, 25.0) |
| *Mtb* | 4.2 (1.6, 11.5) | *Mtb* | 6.6 (3.6, 17.9) |
| *M. cat* | 3.7 (0.0, 16.4) | RSV | 5.0 (0.0, 17.9) |
| Para | 3.3 (0.0, 11.5) | Para | 3.4 (0.0, 14.3) |
| Adeno | 2.8 (0.0, 11.5) | Rhino | 2.2 (0.0, 14.3) |
| **Top 10** | **81.8 (62.3, 95.1)** | **Top 10** | **86.7 (67.9, 100)** |

Abbreviations: Adeno = Adenovirus; CMV = Human cytomegalovirus; EF = Etiologic fraction; Entrb = Enterobacteriaceae; *Hi* non-b = Non-type b *Haemophilus influenzae*; HMPV = Human metapneumovirus A/B; *M. cat* = *Moraxella catarrhalis*; *Mtb* = *Mycobacterium tuberculosis*; NFGNR = Non-fermentative Gram-negative rods; *P. jirov* = *Pneumocystis jirovecii*; Para = Parainfluenza virus; PCV13 = 13-valent pneumococcal conjugate vaccine; Rhino = Human rhinovirus; RSV = Respiratory syncytial virus A/B; *S. aur* = *Staphylococcus aureus*; *S. pneu* Non-PCV13 = Non-13-valent PCV type *Streptococcus pneumoniae*; *S. pneu* PCV13 = 13-valent PCV type *Streptococcus pneumoniae*.

Other Strep includes *Streptococcus pyogenes* and *Enterococcus faecium*. NFGNR includes Acinetobacter species and Pseudomonas species. Enterobacteriaceae includes *E. coli*, Enterobacter species, and Klebsiella species, excluding mixed Gram-negative rods.

Radiologically-confirmed defined as consolidation and/or other infiltrate on chest radiograph.
